# Family Satisfaction in a Medical College Multidisciplinary Intensive Care Unit (ICU)-How Can We Improve?

**DOI:** 10.5005/jp-journals-10071-23122

**Published:** 2019-02

**Authors:** Sowmya Madihalli JanardhanIyengar, Rangalakshmi Srinivasan, Bhaskar Murthy Venkateshmurthy, Yeshaswini Katari, Sahajananda Hiremathada

**Affiliations:** 1,2,5 Department of Anesthesiology and Critical Care, Rajarajeswari Medical College and Hospital, Bengaluru, Karnataka, India; 3,4 Department of Anesthesiology and Critical Care, Rajarajeswari Medical College and Hospital, Bengaluru, Karnataka, India

**Keywords:** Family satisfaction, Intensive care, Questionnaire

## Abstract

**Background and Aims:**

In recent years, patient and family-centered implications are being recognized as important outcome measures and one of the quality indicators of health care system worldwide. Most of the Intensive Care Unit patients cannot make decisions themselves, accordingly family members are surrogate decision-makers and judges of care quality. This study was conducted as a prospective observational study using Family Satisfaction-Intensive Care Unit questionnaire to ascertain the level of family satisfaction of care and their involvement in the decision making process of their patient's treatment.

**Materials and methods:**

The study was conducted over 3 months with 100 family members by FS-ICU questionnaire survey method. After 48 hours of ICU admission, the questionnaire was administered to an eligible family member by a resident who was not involved in the treatment of the patient, in a language understood by them (English/Kannada). Each question was scored using 5 point Likert response Scale and the scores were transformed into 0 (least satisfied) to100 (most satisfied) scale.

**Results:**

Satisfaction with overall care was 65.31±23.62 (FS-ICU/Care). Satisfaction with decision making process was 73.06±22.154 (FS-ICU/ DM). Individual factors which contributed to lower scores were management of pain and agitation of the patient, waiting room atmosphere and emotional support.

**Conclusion:**

This study identified the individual factors which contributed to the high and low satisfaction scores. With this baseline data as reference, there is scope to enhance the aspects of quality care for patients and their family members.

**How to cite this article:**

JanardhanIyengar SM, Srinivasan R *et al*. Family Satisfaction in a Medical College Multidisciplinary Intensive Care Unit (ICU)-How Can We Improve? Indian J of Crit Care Med 2019;23(2):83-88.

## INTRODUCTION

Traditionally, the efficiency of critical care services has been examined by the parameters like mortality, length of stay, outcome and the residual morbidity. In recent years, quality of care has become a central issue in healthcare systems^1^. Betterment of intensive care unit (ICU) quality services involve strategies like providing health care according to evidence based medicine, applying latest guidelines, institutional protocols and following safety and risk management procedures. Monitoring and assessment of these approaches are essential^2^. The distinctive nature of critically ill patients is that they may not attain the desirable health status completely or their final outcome is uncertain. Providing quality care in such circumstances includes family satisfaction with holistic care as an important domain^1^.

Since most ICU patients cannot make decisions themselves due to the varying level of consciousness and the unpredictable severity of their condition, family members who are actively involved in the care process are the surrogate decision-makers. These members are the apt judges of quality of care^2^. In the critical care setting, overall satisfaction with care has to encompass the family. However, satisfaction of care doesn't include the needs of the patients and families which represent separate constructs^3^.

The very nature of critical illness in a family member places their family at a higher risk of developing anxiety, depression, and post-traumatic stress disorder that is post-intensive care syndrome-Family (PICS-F). PICS-F is profoundly affected by the quality of the communication by the medical team^4,5^.

In general, expectations of care, information provided, communication, hospital infrastructure including patient- and family-related factors play a role in family satisfaction with ICU care. Family satisfaction is also related to the family being provided with clear and accurate information because this enables them to actively participate in the decision-making process^2^. Amongst the several tools available for evaluating family satisfaction, we chose ‘family satisfaction in the intensive care unit’ (FS-ICU) questionnaire, a validated score which is accepted worldwide.

### FS-ICU

Heyland and Tranmer developed FS-ICU questionnaire in 2001. It comprises two conceptual domains (1) Satisfaction with care (2) Satisfaction with decision making. This elaborate questionnaire was reduced to 24 multiple choice questions with a five-point Likert response scale in 2007. The questionnaire has been successfully used in a multiple-center study across Canada, indicating good potential for extended use. In addition to studying ‘surrogate decision making’, many quality improvement initiatives have been implemented in the Intensive care set up with this feedback^6^. More recently, FS-ICU was used by the American College of Chest Physicians in a multiple-center intervention study. It focuses on family satisfaction and communication, assesses satisfaction with decision making. These two domains are central to overall family satisfaction with ICU care^6,7^. The questionnaire is accessible online^8^.

With this intent we conducted a prospective observational study on family satisfaction with care of the patients in our intensive care unit using FS-ICU questionnaire. Our objectives were

- To know the level of family satisfaction of overall care
- To ascertain their satisfaction concerning involvement in the decision making process

## MATERIALS AND METHODS

### Study Design

We performed an observational study in patients admitted in our multidisciplinary ICU. Our study population consisted of identified family members (next to kin or decision makers) of adult patients who had been in ICU (MICU/SICU/RICU) for at least 48 hours. The person should have visited the patient often during his/her stay. Patients who had been admitted for less than 48 hours, or family members who may not have comprehended the questionnaire secondary to cognitive, psychiatric or cultural issues and age <18 years were not included.

After obtaining institutional ethical committee clearance, an informed consent was obtained from eligible family members. At least 48 hours after the patient's admission, but before discharge, the questionnaire was administered to the family member by an anesthesia resident who was not involved in the treatment of the patient. It was rendered in a language understood by them (English/Kannada). The study was conducted over 3 months with 100 family members.

The FS-ICU questionnaire has two parts.

1. FS-ICU/Care comprising 14 questions
2. FS-ICU/Decision making comprising 10 questions

Three open-ended questions about the strengths and weakness of our ICU Team and the services were included.

Each question was scored from 1 to 6. 1 being excellent, 2, 3, 4, 5 indicating very good, good, fair, poor, respectively and the score 6 denoting N/A (not applicable) using 5 point Likert response scale. The scores were then transformed into a 0-100 scale. On this scale, 0 is least satisfied and 100 is most satisfied.

Total FS-ICU scores were calculated by averaging individual items, and would range from 0 to 100, with 100 representing highest satisfaction. Scores on the subscales “satisfaction with care” and “satisfaction with decision-making” were also calculated. The third one -a total instrumental score FS-ICU/total for overall ICU experience was calculated by taking the mean of the two satisfaction scores.

Scores above 70 were generally considered satisfactory. Higher scores indicated greater satisfaction while scores below 70 were considered less satisfactory. Factors contributing for both high and low scores were recorded. Responses to the open ended questions were also noted.

### Statistical Analysis

Statistical Methods: Descriptive and inferential statistical analysis has been carried out in the present study^9–11^.

Results on continuous measurements are presented in Mean ± SD (Min-Max) and results on categorical measurements are presented in Number (%).

Statistical software: The Statistical software SPSS 18.0 (IBM Corporation), and R environment ver.3.2.2 (R Development Core Team) were used for the analysis of the data. Microsoft Word and Excel were used to generate graphs, and tables.

## RESULTS

Satisfaction Score was 69.18±22.88 (FS-ICU/Total). Sub scores-Satisfaction with overall care was 65.31±23.62 (**FS-ICU/Care**) and satisfaction with decision making process was 73.06±22.154 (**FS-ICU/DM**) (Table 1).

### Descriptive statistics-Mean Scores 0-100

**Graph 1:**
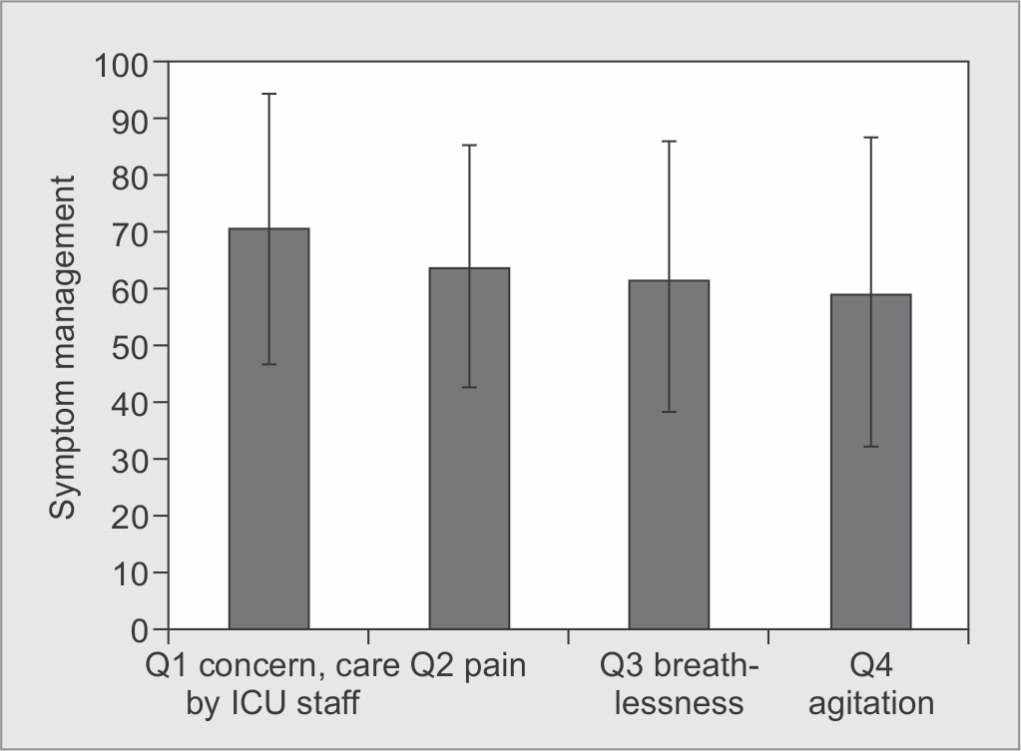
Satisfaction scores for areas-concern, care by ICU staff, symptom management of pain, breathlessness and agitation: Satisfaction score for management of pain was 64.13±21.21, breathlessness was 62.00±23.38 and for agitation, it was 59.66±27.61

**Graph 2:**
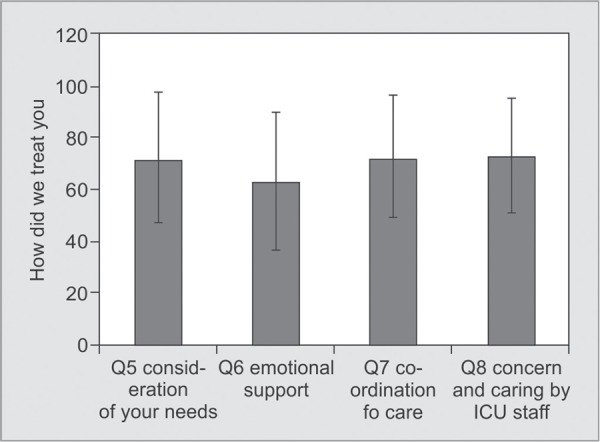
Satisfaction scores for “How did we treat the family member” (Respondent): Satisfaction score for consideration of their needs was 72.25 ± 24.85, for team work (coordination of care) −72.73 ± 23.18, for courtesy, respect (concern and caring) −73.74 ± 22.12. Satisfaction score for providing emotional support was 63.51 ± 26.08

**Graph 3:**
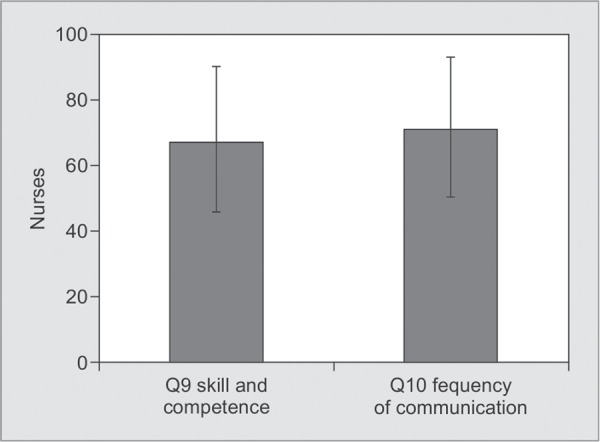
Satisfaction with nurses' skill, competence and frequency of communication: Satisfaction score for skill and competence of nurses was 67.82±23.08 and for the frequency of communication with them was 71.21±22.12.

**Graph 4:**
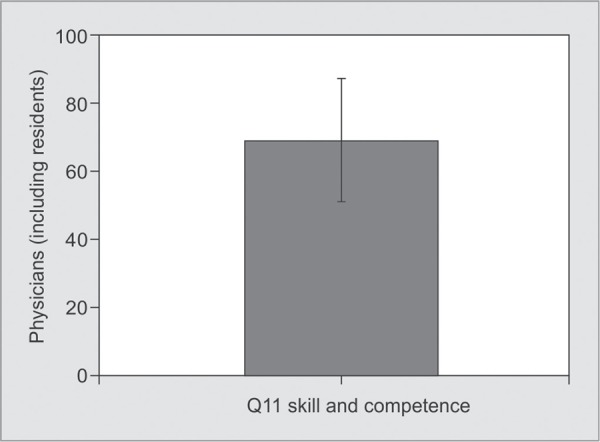
Satisfaction with skill and competence of the physicians (ICU doctors including residents): The score for care shown by doctors was 79.00±20.63

**Graph 5:**
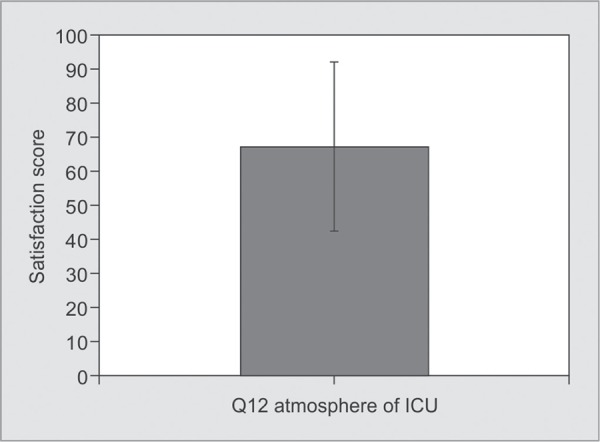
Satisfaction score with atmosphere of ICU: The score for the atmosphere of ICU was 67.25±24.54

**Graph 6:**
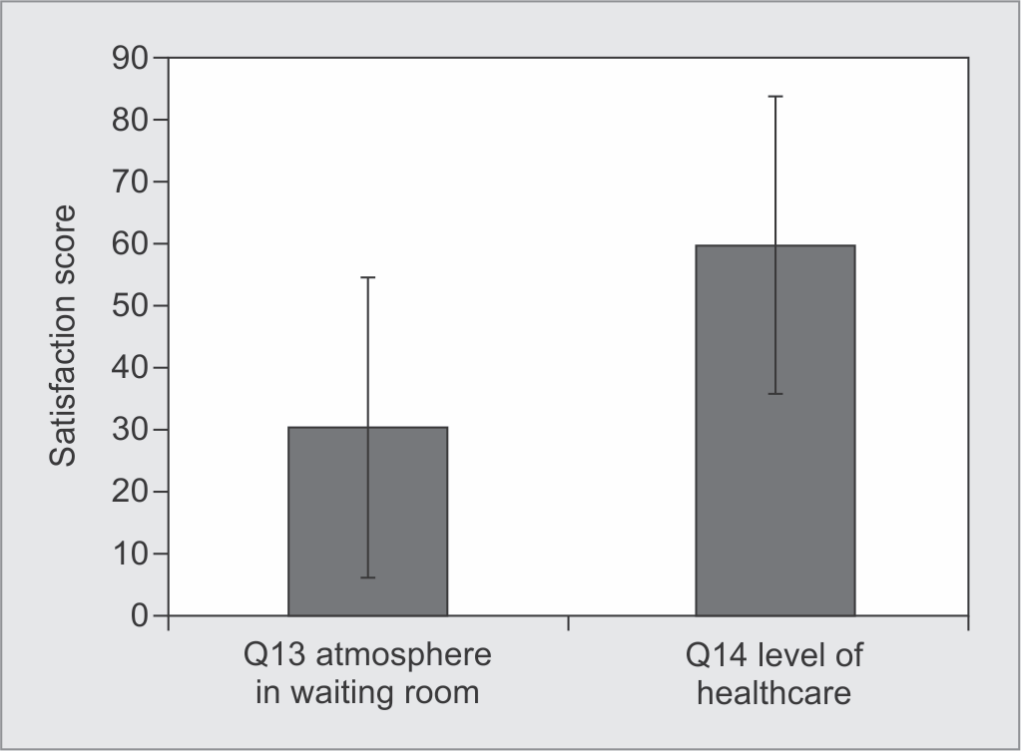
Satisfaction score for atmosphere of ICU waiting room and level of healthcare patient received in the ICU: Satisfaction score for ICU waiting room was 30.41±24.27 and overall satisfaction with the level of healthcare received by the patient, it was 60.00±23.84

*Part 1:* Satisfaction with Care (Table 2 and Graphs 1 to 6)

**Graph 7:**
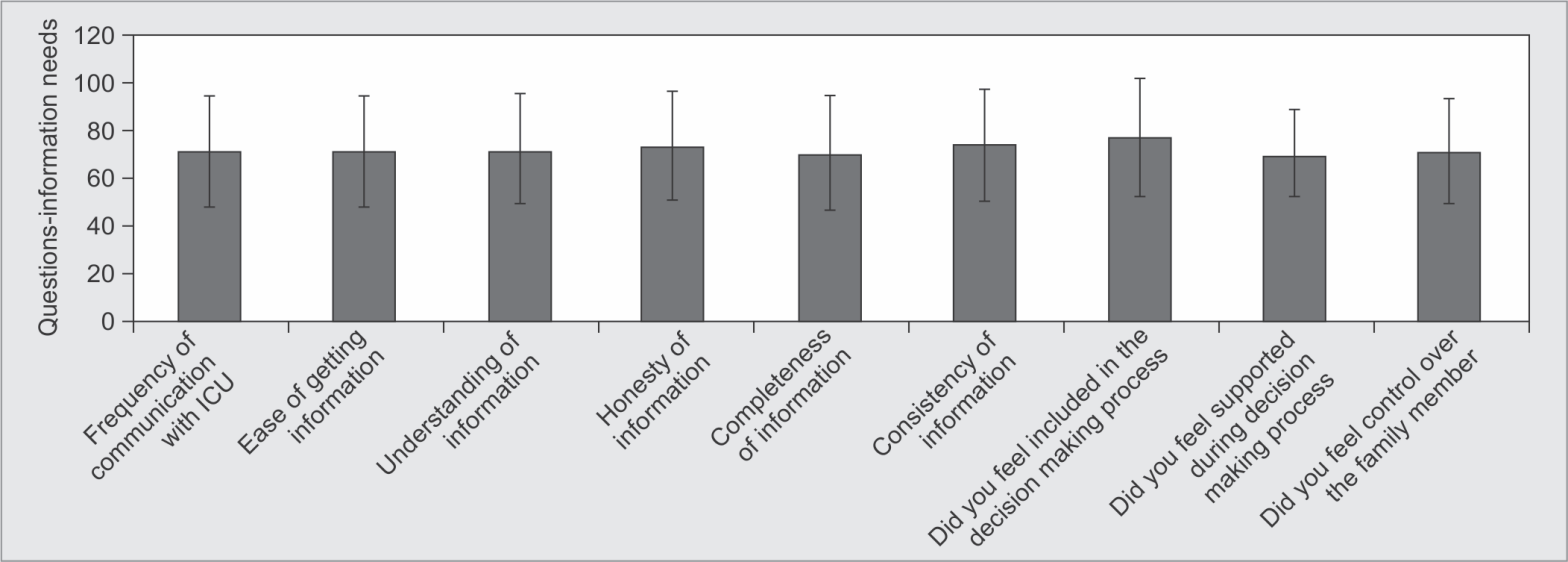
Satisfaction with information provided and decision making process

*Part 2:* Family Satisfaction with Decision Making (Tables 3 and 4, Graph 7)

## DISCUSSION

Families of the critically ill patients have to face a stressful environment. For a majority of them it is an entirely new and frightening experience where they have to fall back wholly on the account given by the medical team with regards to the condition of the person in ICU in uncertain circumstances. This, added to the possible mortality of their relative makes them more prone to develop PICS-F(Post Intensive care syndrome-Family). It follows that the communication skills of the counseling team/medical team play a great role in the prevention of PICS-F.

In our study, 68% of the respondents were facing this situation for the first time. Their average age was 36.29 years (21-75 years) (Table 1).

Our mean scores (±SD) for FS-ICU/Total, FS-ICU/care and FS-ICU/ decision making were 69.18±22.88, 65.31±23.62, and 73.06±22.154, respectively.

These were low when compared to Canadian data (82.9±14.8, 83.5±15.4, 82.6±16.0) which was from a multiple center study^12^.

**Table 1:** Baseline characteristics of respondents: 68% respondents did not have any experience about critical illness and ICU environment and were facing it for the first time.

Average age	36.29 years (21-75 years)
Gender	Male: 64 Female: 36
Lived with the patient or not	74 lived with the patient
	2 visited several times a week
	8 visited once a week 11 visited every month 5 visited once a year
Place of residence	55 lived in the same city 45 lived outstation
Previous ICU experience as a family member	32 involved 68 not involved

**Table 2:** Satisfaction with care of patients

*Questions*	*Min-Max*	*Mean ± SD*
Symptom management		
• Q1 concern, care by ICU staff	0.00-100.00	70.75±23.85
• Q2. Pain	0.00-100.00	64.13±21.21
• Q3. Breathlessness	0.00-100.00	62.00±23.38
• Q4. Agitation	0.00-100.00	59.66±27.61
How did we treat you		
• Q5. consideration of your needs	0.00-100.00	72.25±24.85
• Q6. Emotional support	0.00-100.00	63.51±26.08
• Q7. Co-ordination of care	0.00-100.00	72.73±23.18
• Q8. Concern and caring by ICU staff	0.00-100.00	73.74±22.12
Nurses		
• Q9. Skill and competence	0.00-100.00	67.82±23.08
• Q10. Frequency of communication	0.00-100.00	71.21±22.12
Physicians (including residents)		
• Q11. Skill and competence	0.00-100.00	79.00±20.63
The ICU		
• Q12. Atmosphere of ICU	0.00-100.00	67.25±24.54
The waiting room		
• Q13. Atmosphere in waiting room	0.00-100.00	30.41±24.27
• Q14. Your satisfaction with Level of healthcare	0.00-100.00	60.00±23.84

The results from a study in an ICU, in Hong Kong, by SM Lam *et al.*^12^, on 961 families, were 78.1±14.3 (FS-ICU/Total), 78.0±16.8 (FS-ICU/Care) and 78.6±13.6 (FS-ICU/DM). This survey showed high satisfaction scores that were similar to results reported around the world.

A German study with 215 families, reported their mean FS-ICU/ Total, FS-ICU/Care, and FS-ICU/DM as 78.3 ± 14.3, 78.6 ± 14.3, and 77.8 ± 15.616, respectively^13^.

In a Swiss study, on 996 family members from multiple centers, the summary scores were 78 ± 14 (FS-ICU/Total), 79 ± 14 (FS-ICU/ Care) and 77 ± 15(FS-ICU/Decision making)^14^. An American multisite cross-sectional study with 1290 respondents from 15 hospitals in western Washington State, achieved scores of 76.6 ± 20.6, 77.7 ± 20.6, and 75.2 ± 22.6, respectively^15^.

The Canadian study data showed a significantly better result in most items and in the total and subscale scores, indicating room for further improvements in other centers. This might be explained by cross-cultural (different expectations from families), as well as administrative differences (nurse-patient and doctor-patient ratios)^12^. Both the above countries are developed nations where healthcare is free. Their per capita income precludes the average person from financial worries for healthcare. Canada has a fully state sponsored healthcare. Better educational background also leads to better awareness and understanding of the illnesses. India despite great strides in several spheres of development needs to percolate comprehensive and advanced health care to grass root level. Our hospital and ICU is located in a semirural area, where health care is subsidised and the majority of patients are from a low educational and socioeconomic strata of society. The other significant factor in patient profile was exhaustion of their funds due to their arriving at the hospital mostly as a last resort after several referrals. Notwithstanding the above limitations, we decided to survey the satisfaction scores.

**Table 3:** Information needs and involvement in decision making process: Out of 100 respondents, 80 felt they were given adequate time for addressing their concerns and answering their questions.

*Questions-information needs*	*Min-Max*	*Mean ± SD*
Frequency of communication with ICU doctors	0.00-100.00	71.00±23.23
Ease of getting information	0.00-100.00	71.46±23.15
Understanding of Information	0.00-100.00	72.00±23.11
Honesty of information	0.00-100.00	73.74±22.41
Completeness of information	0.00-125.00	70.25±24.28
Consistency of information	0.00-100.00	74.23±23.39
Did you feel included in the decision making process	0.00-100.00	77.00±25.05
Did you feel supported during decision making process	0.00-100.00	69.95±18.89
Did you feel control over the family member	0.00-100.00	71.00±22.11

There are very few Indian studies on family satisfaction using a validated FS-ICU questionnaire for comparison.

In a survey by Venkataraman *et al*.^16^, on 200 family members using modified and adapted family satisfaction questionnaire, 94.5% of them were satisfied with the overall care, 90.5% showed a positive response with Staff Interaction. 84.5% of the respondents were satisfied with the medical counselling and 80.5% with the facilities. Lower satisfaction scores were obtained with their visiting hours policy. (60.5% of respondents were satisfied with the visiting hours)

The family satisfaction scores regarding ‘consideration of their needs, concern and caring by the ICU staff’ were 72.25±24.85 and 73.74±22.12 (Table 2) respectively, which was satisfactory. The score for ‘Skill and competence of Physicians including residents’ was 79.00±20.63(Table 2), which was very good. We could surmise that the medical team did not lack skills and sincerity.

Individual factors which contributed to our lower scores compared to international standards were symptom management of pain (64.13±21.21), breathlessness (62.00±23.88), agitation (59.66±27.61), emotional support of the family (63.51±26.08), skill and competence of nurses (67.82±23.08), atmosphere in ICU (67.25±24.54), atmosphere of waiting room for family members (30.41±24.27) (Table 2).

*Agitation management:* Sedation protocol of our ICU required withholding morning sedation in order to assess the consciousness and sedation scale. Of late, evidence based medicine suggests a light or moderate sedation is preferred to deep levels to reduce the duration of mechanical ventilation and length of hospital stay. It has always been a challenge to strike a balance between achieving a moderate to light sedation whilst preventing agitation^17,18,19^. Management of agitation thereby warranted urgent attention.

*Atmosphere in the ICU:* Most of the respondents felt the patients were uncomfortable with the lower temperatures of ICU. One explanation for this again could be the social background of patients. Another feedback we received was regarding housekeeping. The respondents felt that it could be better.

**Table 4:** Response for the open ended questions

*Questions*	*Description of the responses*	*Number of responses*
Comments on things we did well	Acknowledged the Doctors and nurses team work and the treatment	88
Suggestions on making the care provided better	Reduce the number of visitors and frequency of visits	6
	To have separate ICU pharmacy in the vicinity of ICU	5
	To increase the number of housekeeping staff	5
	Financial assistance/support for very poor patients	4
Suggestions that may be helpful to our staff	Nurses and housekeeping staff to be more polite	6

Our score for the question on atmosphere in the waiting room was (30.41±24.27) (Table 2). The ICU is located on second floor serviced by a single elevator. The waiting room for family members is a common lounge with rest room on the ground floor. Public address system is used to summon them when required. The relatives would be called in for regular counseling sessions with the ICU team. Other reasons such as to report change in the medical status, any medications to be procured or if the family needed to clarify their apprehensions about the patient's condition also warranted an interview with the medical team. Frequent visits to the second level were imperative. All of these explained why family members felt distanced from their relative in ICU. Several respondents also felt the need for their private space in their difficult times.

*Skill and Competence of Nurses:* The recommended nurse patient ratio in ICU is 1:1 or 1:2 depending on the stability of the patient (1:1 if patient on mechanical ventilation)^20^. This ensures complete care of the patient. Our ICU had a ratio of 1:3 for non-ventilated patients while for ventilated patients we maintained 1:1 ratio. There were few open-ended questions in the questionnaire which revealed that few family members felt the nurses were harsh and rude while communicating to them (Table 4).

The scores for Decision making process and the need of information were satisfactory (73.06±22.154) (Table 3). We presume that we provided the needed information to the family members during counseling, gave them adequate time for decision making during the critical periods of their ward in our ICU and adequately involved them in the decision making process. These factors are admittedly important in reducing the risk of developing Post Intensive care syndrome-Family (post-traumatic stress disorder)^4,5^.

In the study by Lam *et al.*^12^, a Performance-Importance plot was done. The factors which had greater regression weights but performed less satisfactorily were identified. The following factors required urgent attention: atmosphere of ICU, atmosphere of ICU waiting room, agitation management, and satisfaction with level of health care. Many studies have identified ICU environment as the factor that affects satisfaction^15,21,22^. Our study results were consistent with the results of the above study.

These results were discussed with the Management Committee and Human Resources Department of our hospital and we decided to refurbish ICU waiting room and provide facilities, with an increase in the nurse-patient ratio for providing efficient nursing services. A team comprising doctors and nurses was deputed to train them regularly in their deficiencies. A protocol was made to get regular feedback from the family members and examine it once in 2 weeks. Training sessions on communication skills were planned to improve the quality of counseling by care givers (including residents).

### Limitations of the Study

The questionnaire was translated verbatim in the local language. It wasn't modified according to the regional/cultural needs.

Association with the basic demographic characteristics of the respondents wasn't established (age, sex, education level, socioeconomic state, or patient's severity of disease). Family response rate was not considered. Ours was a one time response survey when the patient was still in the ICU under our care and not the final one after the patient was discharged home or if the patient did not survive.

## CONCLUSION

Family satisfaction is one of several quality indicators of medical care. Our study, using FS-ICU questionnaire, is one of the few surveys done in India. There is enormous scope for improvement in providing quality care to our ICU patients and facilities for members of their who are going through disquieting and uncertain circumstances.

It is desirable that each hospital conduct a family satisfaction feedback for self-assessment and thereon improve in the specified areas.

### Implications in Clinical Practice

The identified individual factors give directions for further revamping our service. It also serves as a baseline data for regular feedback comparisons and self -evaluation of our ICU efficacy.

### Future Scope

Development of a modified FS-ICU questionnaire, suitable for the Indian patient population and their family based on the cultural and regional differences and in local languages and its validation.
